# Maternal Folic Acid Supplementation, Perinatal Factors, and Pre-Adolescent Asthma: Findings from the Healthy Growth Study

**DOI:** 10.3390/nu17182989

**Published:** 2025-09-18

**Authors:** Eva Karaglani, Maria Michelle Papamichael, Matzourana Argyropoulou, Dimitra-Irinna Vitoratou, Costas Anastasiou, Mehak Batra, Yibeltal Bekele, Bircan Erbas, Yannis Manios, George Moschonis

**Affiliations:** 1 Department of Nutrition and Dietetics, School of Health Science and Education, Harokopio University of Athens, 17671 Athens, Greece; ekaragl@hua.gr (E.K.); margyropoulou@hua.gr (M.A.); ivitoratou@hua.gr (D.-I.V.); acostas@hua.gr (C.A.); manios@hua.gr (Y.M.); 2 European Centre for Obesity, Harokopio University, 17671 Athens, Greece; 3 Department of Food, Nutrition and Dietetics, School of Allied Health, La Trobe University, Bundoora Campus, Melbourne, VIC 3086, Australia; sassipap@hotmail.com; 4 Department of Public Health, School of Psychology and Public Health, La Trobe University, Bundoora Campus, Melbourne, VIC 3086, Australia; m.batra@latrobe.edu.au (M.B.); y.bekele@latrobe.edu.au (Y.B.); b.erbas@latrobe.edu.au (B.E.); 5 Institute of Agri-Food and Life Sciences, Hellenic Mediterranean University Research Centre, 72100 Crete, Greece

**Keywords:** asthma, environment, epigenetics, maternal folic acid supplementation, pre-adolescents, pregnancy, perinatal

## Abstract

**Background:** While the importance of folic acid supplementation during pregnancy in the prevention of neural tube defects in offspring is well established, its potential role in pediatric asthma development remains unclear, with limited evidence to date. **Objective:** To identify perinatal and environmental factors that modify the association between maternal folic acid intake and pre-adolescent asthma. **Methods:** Cross-sectional analysis of the Healthy Growth Study that consisted of 2332 pre-adolescents (mean age 11 years; asthma *n* = 451); 50% boys attending elementary schools in Greece. Questionnaires were used to collect data on sociodemographic, perinatal, and environmental characteristics as well as asthma prevalence and maternal folic acid supplementation during pregnancy (trimesters 1, 2, and 3). Logistic regression models explored the association between maternal folic acid supplementation and pre-adolescent asthma, accounting for perinatal and environmental exposures. **Results:** Adjusted regression models showed that maternal folic acid supplementation during the third trimester was associated with 34% increased odds of pre-adolescent asthma. Stratified analyses per perinatal and environmental factors revealed significantly higher asthma odds with folic acid supplementation during the second and third trimesters among pre-adolescents born < 37 weeks; non-smoking mothers; in pre-adolescents attending schools of low socioeconomic level; and in neighborhoods having less traffic and more parks. Contrastingly, in appropriate for gestational age (AGA), an infant’s first-trimester supplementation increased asthma odds. **Conclusions:** Maternal folic acid supplementation, particularly in later trimesters, was modestly associated with increased odds of pre-adolescent asthma, modified by perinatal and environmental factors. Future research should explore whether continued folic acid supplementation beyond the first trimester carries differential risks or benefits in asthma.

## 1. Introduction

The high incidence of asthma during the pediatric years continues to baffle the medical community. Global trends substantiate that about 9% of children and 11% adolescents live with asthma [1]. Asthma, a chronic inflammatory lung disease, is defined by characteristic symptoms of wheezing, cough, chest tightness, and shortness of breath of varying severity and intensity [2]. On a worldwide scale, this lung disorder is ranked among the top 20 chronic pediatric diseases for disability-adjusted life years [3], exerting a serious threat to children’s well-being [3]. Furthermore, it contributes to increased morbidity, causing a physical and mental toll [4]. Asthma-related mortality rates are highest in countries with a low and lower-middle sociodemographic index [5] due to underdiagnosis and inadequate treatment [6]. Even in high-income countries, population-specific differences in asthma risk and management have been observed, underscoring the need for localized evidence [7]. In this context, studying the Greek pediatric population is particularly important, given potential variations in genetic predisposition, diet, healthcare access, and environmental exposures that may influence asthma outcomes [8,9].

This condition is one of the main reasons for children’s school absenteeism [10], poor academic performance [11], and sleep deprivation [12]. In general, asthma results in decreased quality of life for the patient and a financial burden for the family and national healthcare system due to the need for emergency visits, hospitalizations, and medication use [13]. Disturbingly, the World Health Organization (WHO) predicts that by the year 2030, 400 million children, adolescents, and adults will suffer from asthma globally [14]. Currently, there is no remedy for asthma. Medication remains the gold standard for managing bronchial symptoms [2]. However, asthma control in pediatric populations remains suboptimal due to non-compliance with therapy [15] and, in some cases, limited access to healthcare resources and inadequate availability of pulmonary function tests and medications [16]. Therefore, identifying risk factors to prevent asthma onset is critical to curbing its rise and burden in pediatric populations.

Over the last decade, increasing attention has been given to the impact of in utero life on offspring health. The infamous Barker theory proposes that the intrauterine environment that the growing fetus is directly exposed to, including the maternal diet, plays a central role in the development of future chronic diseases in adulthood, including allergies [17]. Maternal exposures to nutritional and non-nutritional stressors during pregnancy can be transferred to the fetus via the placenta, shaping fetal immune responses during critical periods of development [18]. It is believed that environmental insults could cause epigenetic alterations, including DNA methylation, histone modifications, and non-coding RNAs, impacting gene expression, favoring asthma development in progeny [19]. The concept of developmental plasticity or sensitivity to environmental stimuli, the phenomenon by which a specific genotype could initiate a diversity of physiological or morphological states in response to different environmental conditions during critical stages of development, is indeed intriguing [17] and warrants further investigation. This cascade could prime and trigger allergic sensitization and lead to allergic disorders, including asthma in the pediatric years [18].

A plethora of systematic reviews and meta-analyses have documented the significance of maternal diet quality promoting favorable birth outcomes [20,21]. Specifically, poor maternal diet quality has been significantly associated with higher odds of low birth weight, small for gestational age (SGA) [20,21], and preterm birth [21]. Emerging evidence also suggests that maternal diet during pregnancy may influence the risk of asthma development in offspring [22]. While folic acid supplementation is well established for its role in preventing neural tube defects in offspring [23], its potential contribution to pediatric asthma remains unclear and warrants further investigation. In particular, there is limited evidence on the effects of folic acid supplementation by trimester of exposure and whether environmental or perinatal factors (e.g., socioeconomic status, preterm birth, or air pollution) may modify this association. Recently, Li et al. conducted a meta-analysis investigating the effect of maternal folic acid supplementation during pregnancy that included 15 studies and over 200,000 children from 1–10 years old [24]. Results showed that exposure to maternal folic acid supplementation during pregnancy was associated with an 11% higher risk of asthma in children (RR  =  1.11; 95% CI  =  1.06–1.17). Along the same line, Yang et al. conducted a systematic review of 18 relevant studies (13 cohort studies; 5 case-control studies) investigating the effect of folic acid intake during pregnancy and childhood asthma [25]. Pooled analysis of data from 253,000 cases (ages ranged from 1 to 12 years) and about 50,000 children with asthma revealed that maternal folic acid intake during the first trimester was associated with 9% higher odds of asthma in offspring (first trimester OR  =  1.09; 95% CI  =  1.05–1.12), 15% higher odds in the third trimester (OR  =  1.15; 95% CI  =  1.04–1.26), and 13% higher odds of asthma for folic acid intake during the whole pregnancy (OR  =  1.13; 95% CI  =  1.10–1.16). Interestingly, the dose–response analysis showed that folic acid supplementation > 581 μg/day increased asthma risk in offspring [25]. Therefore, the potential association between folic acid association during pregnancy, commonly prescribed to prevent neural tube defects, and the risk of asthma in offspring warrants careful consideration.

Despite growing evidence, there is still a gap in understanding how perinatal and environmental factors may modify this association. This study aimed to identify such modifiers that influence the relationship between maternal folic acid supplementation and asthma in pre-adolescent children. We hypothesized that perinatal and various environmental exposures strengthen/predict the association between maternal folic acid supplementation and asthma in pre-adolescence. From a public health point of view, the findings of this study may have relevance in formulating public health strategies and on the implementation of childhood asthma preventive measures, especially in those at high risk for this condition. Early intervention could prevent future occurrences of asthma and alleviate the worldwide burden of this chronic respiratory disease.

## 2. Materials and Methods

### 2.1. Study Design and Population

The present study was a secondary analysis of data from the Hellenic Healthy Growth Study (HGS), which was a 2-year observational study conducted from May 2007 until June 2009 and included a sample of 9–13-year-old schoolchildren attending grades five and six of elementary school situated in the district of Athens, Northern Thessaloniki, Aitoloakarnania and Southern Heraklion, Crete [26,27]. The sampling of municipalities and schools was random, multistage, and stratified according to parental educational level and the total population of pre-adolescent students. Socioeconomic levels (SELs) defined as high, medium, and low were based on data from the Hellenic National Statistical Service of Greece (2001) [28]. Municipalities and schools were selected from these three SEL groups, thus providing a representative sample of the national Hellenic population. Complete details of the study design and methodologies are available in a previous publication [26].

### 2.2. Ethical Considerations

The HGS was conducted in accordance with the Declaration of Helsinki. Ethical approval of the study protocol was obtained from the Hellenic Ministry of Education and Human Ethics Committee of Harokopio University, Athens, Greece (Protocol ID 16/19 December 2006). Upon study enrollment, parents were provided with a detailed letter explaining the study objectives and assessments and were asked to sign a written informed consent form. Overall, 4145 children were eligible to participate, and 2656 were enrolled in the study (64% response rate). For the purpose of this sub-analysis, the study population included 2332 Greek schoolchildren aged 9–11 years, 451 of whom had asthma.

### 2.3. Data Collection

Involvement in the HGS was voluntary. Parents willing to participate were instructed to attend face-to-face interviews scheduled during school hours, or telephone interviews were conducted by rigorously trained researchers. In all schools and districts, standardized questionnaires were used to collate details on sociodemographic characteristics as well as perinatal and environmental exposures (Supplemental Material S1 HGS Parental Questionnaire). More specifically, information included the mother’s age (years), maternal educational level (primary, secondary, tertiary), the child’s age (years) and sex, and the socioeconomic level of the school (lower, medium, higher). The perinatal and environmental characteristics comprised: gestational age (weeks), mode of delivery (Cesarean section; the normal route), gestational diabetes (Yes, No, I don’t know), weight category for gestational age (appropriate (AGA), small (SGA), or large for gestational age (LGA)), exclusive breastfeeding (Yes/No), pre-pregnancy weight (underweight, normal, overweight, obese), pregnancy weight gain (kg), folic acid intake (trimesters 1–3), maternal smoking during pregnancy (Yes/No), passive smoking during pregnancy (Yes/No), neighborhood has parks and areas for exercise (disagree/agree), and neighborhood has too much traffic (disagree/agree).

### 2.4. Asthma Status

The presence of asthma in pre-adolescents was assessed using the International Study on Asthma and Allergy in Childhood (ISAAC) core respiratory questionnaire [29], which assessed asthma prevalence or wheezing in 6–7-year-old children and 13–14-year-old adolescents. For the purpose of this study, a composite variable for ‘asthma’ (yes or no) was created by summing a positive answer of ‘yes’ for these questions.

### 2.5. Maternal Folic Acid Supplementation

Maternal folic acid supplementation throughout pregnancy was self-reported and assessed through the question, ‘Which trimester of pregnancy did your take folic acid supplements (1st, 2nd, or 3rd)?’

### 2.6. Statistical Analysis

Statistical analysis was performed using the Statistical Package for Social Sciences (SPSS, version 24.0, Armonk, NY, USA, IBM Corp). Missing data were handled using listwise deletion. Complete case analysis was conducted, with 2066 participants retained for multivariable regression models. All analyses were two-tailed, and statistical significance was considered at alpha = 0.05. Continuous variables were assessed if they conformed with the normal distribution using the Kolmogorov–Smirnov test and graphically via the P–P plot. Continuous variables are presented as means ± standard deviation (SD) and as medians, minimum/maximum values, and interquartile range (IQR) in the case of skewness. Group differences were evaluated using an independent *t*-test for normally distributed variables and, otherwise, by the non-parametric Mann–Whitney and Chi-square tests. Associations between maternal folic acid supplementation (independent variable) and pre-adolescent asthma (dependent) were assessed using logistic regression models stratified by child’s sex, socioeconomic level, gestational age, infant weight for age, and maternal smoking during pregnancy. Adjustments were made for potential perinatal and environmental characteristics based on risk factors for asthma from the literature and by analyzing directed acyclic graphs [30] (Figure S1)—namely, child’s sex, maternal education level, exclusive breastfeeding, passive smoking during pregnancy, pre-pregnancy weight, maternal age, gestational diabetes, pregnancy weight gain, and mode of delivery [31,32,33].

To evaluate potential effect modification, stratified analyses were conducted by child’s sex, gestational age, weight for gestational age, maternal smoking during pregnancy, neighborhood characteristics, and school socioeconomic level. These factors were selected based on previous evidence of their potential to influence asthma risk and observed differences across exposure and outcome groups in our data [34,35,36].

## 3. Results

In this cross-sectional analysis of 2332 pre-adolescents, those with asthma (*n* = 451, 19.3%) had a slightly lower mean age (11.10 ± 0.62 years) compared with those without asthma (11.18 ± 0.68 years, *p* = 0.040). A significantly greater proportion of males (58.3%, *n* = 263) was observed among pre-adolescents with asthma compared with those without asthma (47.4%, *n* = 892, *p*-value < 0.001). There was a significant association between the school socioeconomic level and the pre-adolescent asthma status (*p* = 0.017). A greater proportion of pre-adolescents with asthma was observed at the higher school socioeconomic level (46.8%, *n* = 211) compared with pre-adolescents without asthma (39.5%, *n* = 743), whereas lower proportions of pre-adolescents with asthma were observed in lower (29.6%, *n* = 133) and medium (23.6%, *n* = 106) school socioeconomic levels compared with those without asthma (lower = 26.5%, *n* = 498; medium = 34%, *n* = 638). A slightly lower proportion of pre-adolescents with asthma resided in neighborhoods with high traffic (64.5%, *n* = 236) compared with those without asthma (68.9%, *n* = 1062, *p* = 0.042). Furthermore, a greater proportion of pre-adolescents with asthma (29.7%) were born to mothers who were exposed to passive smoking during pregnancy compared with those without asthma (25.1%, *p* = 0.047). Additionally, a higher percentage of pre-adolescents with asthma were delivered via Cesarean section compared with those without asthma (33.7%, *n* = 152 vs. 27.4%, *n* = 515, *p* = 0.008). Although a slightly higher prevalence of asthma was noted among pre-adolescents whose mothers took folic acid during pregnancy, compared with those without asthma, this difference did not reach statistical significance across the first (18.9% vs. 15.3%, *p* = 0.059), second (25.1% vs. 20.9%, *p* = 0.054), or third trimester (23.6% vs. 19.6%, *p* = 0.059). No significant differences in asthma prevalence were observed concerning maternal education level, neighborhood park availability, gestational age, weight for gestational age, exclusive breastfeeding, or maternal smoking during pregnancy (*p* > 0.05 for all) (Table 1).

Regression models showed significant associations between maternal folic acid supplementation during the third trimester of pregnancy and increased odds of pre-adolescent asthma. More specifically, adjusted odds ratios (aORs) indicated 32% higher odds of asthma with supplementation during the first trimester (aOR = 1.32; 95% CI: 0.99, 1.76; *p* = 0.063), 30% higher during the second trimester (aOR = 1.30; 95% CI: 0.99, 1.68; *p* = 0.051), although non-significant, and 34% higher during the third trimester (aOR = 1.34; 95% CI: 1.03, 1.75; *p* = 0.030). Stratified analyses revealed that these associations were significant among male pre-adolescents for the first- and second-trimester pregnancy supplementation, with the highest odds observed for first-trimester pregnancy supplementation (aOR = 1.57; 95% CI: 1.07, 2.29; *p* = 0.018). No significant associations were found among females (Table 2).

Table 3 presents the association between maternal folic acid supplementation during pregnancy and pre-adolescent asthma stratified by perinatal characteristics. Among children born at term (≥37 weeks), folic acid supplementation during pregnancy was not associated with pre-adolescent asthma. However, in pre-adolescents born prematurely, (<37 weeks), a significant association was observed for third-trimester supplementation (aOR = 1.82; 95% CI: 1.01, 3.27; *p* = 0.046). When stratified by weight for gestational age, AGA infants exhibited a significant association between first-trimester folic acid intake and asthma (aOR = 1.41; 95% CI: 1.02, 1.95; *p* = 0.036). No significant associations were found among SGA or LGA infants across all trimesters.

Table 4 presents the association between maternal folic acid supplementation during pregnancy and pre-adolescent asthma stratified by environmental and external factors. Among non-smoking mothers, second-trimester supplementation was associated with an aOR of 1.44 (95% CI: 1.08, 1.91; *p* = 0.012), and third-trimester supplementation with an aOR of 1.52 (95% CI: 1.14, 2.02; *p* = 0.005). No significant associations were observed among mothers who smoked during pregnancy. Stratification by school SEL indicated that in the low SEL group, maternal folic acid supplementation throughout pregnancy was associated with pre-adolescent asthma, with the highest odds observed when the supplementation occurred in the third trimester with an aOR of 2.02 (95% CI: 1.17, 3.49; *p* = 0.011. No significant associations were found in the medium or high SEL groups. Regarding neighborhood characteristics, in areas reported to have less traffic and more parks, third-trimester supplementation was linked to an aOR of 1.73 (95% CI: 1.07, 2.82; *p* = 0.026) and an aOR of 1.52 (95% CI: 1.00, 2.30; *p* = 0.047), respectively.

## 4. Discussion

The present study endeavored to identify perinatal and environmental exposures that contribute to the association of maternal folic acid supplementation during pregnancy and pre-adolescent asthma. Our analysis found that folic acid supplementation during the third trimester was associated with increased odds of asthma in pre-adolescents. Stratified analyses revealed stronger associations among male children, those born preterm, children of non-smoking mothers, those attending schools in low socioeconomic areas, and those living in neighborhoods with less traffic and more parks. Additionally, among AGA infants, first-trimester supplementation was significantly associated with increased asthma odds.

Descriptive statistics revealed that, in comparison with healthy pre-adolescents without asthma, those with asthma belonged to the male sex, attended schools of lower socioeconomic level, were delivered via Cesarean section, and were born to mothers exposed to passive smoking during pregnancy. These features are consistent with data from previous studies [24,25,37,38,39,40]. Clinical and animal studies have demonstrated gender disparities in asthma development [37,38,41], with boys exhibiting higher susceptibility to wheezing and asthma than girls before puberty, after which the prevalence pattern is reversed [37,38].

An outstanding finding of the current study, data analysis revealed that maternal folic acid supplementation during pregnancy, particularly during the third trimester, was associated with increased asthma odds during pre-adolescence. Epigenetics may explain the impact of external environmental insults combined with genetic susceptibility on disease pathogenesis [42]. In this context, in utero nutritional exposures have the capacity to epigenetically modulate genomic regions in offspring and play a critical role in driving immune dysregulation and, as a consequence, amplifying the development of allergic disorders, including asthma [43,44]. The perinatal period offers a window of opportunity for the influence of environmental factors (including nutrition) to be modulated by intrauterine epigenetic programming [44]. This is in line with the Barker theory that environmental events during the first 1000 days of life are pivotal in determining the development of chronic diseases, including allergic disease in later life [45]. Conceptually, fetal plasticity during this critical time of fetal maturation and organ development might explain the association between maternal diet and childhood asthma. In line with the Barker theory, there may be a critical window during the third trimester when prenatal folic acid exposure predisposes the fetus to an asthma phenotype [45]. Folic acid, a methyl donor and key player in the one-carbon cycle, is pivotal for the synthesis of methionine and S-adenosylmethionine (SAM), both of which are essential intermediates for DNA methylation, the synthesis of nucleotides, protein (especially methionine), and in regulating homocysteine levels—a cardiovascular risk factor [44,46]. Hollingsworth et al., in a murine model, demonstrated that a methyl-rich diet during pregnancy caused aberrations in DNA methylation at RUNX3 loci [47,48]. In fact, hypermethylation at RUNX3 loci triggered differentiation of T cells toward a Th2 phenotype that promoted asthma induction in mice. In addition, increased airway hyperreactivity, higher levels of lung lavage eosinophilia, IL-13, and IgE—all characteristic features of asthma pathogenesis—were noted in mice fed a methyl-rich diet vs. a regular diet. Interestingly, mice exposed to a methyl-rich diet postnatally, either during lactation or in adulthood, did not induce an asthma phenotype. Emerging research indicates that epigenetic mechanisms such as gene methylation could play an essential role in childhood asthma through the regulation of genes involved in allergic responses [19]. Epigenetic control of IgE and nitric oxide might promote and maintain airway inflammation, creating a background favoring asthma development [49]. More specifically, hypomethylation of interleukin-6 (IL-6) and nitric oxide synthase-2(NOS-2) was associated with increased fractional exhaled nitric oxide (FeNO), forced expiratory volume in 1 s, and wheezing in 8–11-year-old children [49]. Human and in vitro studies demonstrate that two microRNAs, specifically miR-155 and miR-221, were associated with T-helper 2 (Th2)-derived cytokine inflammation (IL-13) and cellular elements of the immunological response relevant to asthma, including eosinophils, macrophages, and mast cells [50,51,52].

One other epigenetic mechanism worth consideration is that high maternal folate levels during fetal life have been linked with increased histone acetylation of Th2 loci in cord blood CD4+ T cells along with a more transcriptionally permissive chromatin state. Harb et al. used cord blood samples from a cohort of Australian neonates exposed to maternal folate supplementation in utero. It was documented that very high levels of maternal folate were associated with increased histone acetylation (H3/H4) at the GATA 3 gene loci (a Th2-related gene) and IL-9 (Th2-derived cytokine) promoter regions in CD4+ T cells in neonatal cord blood as compared with the low folate group [53]. This study demonstrated an association between folate exposure during gestation and increased histone acetylation corresponding to a more transcriptionally permissive chromatin state in the promoter regions of pro-inflammatory Th2 genes [42].

From another perspective, developments in neonatal immunology suggest that allergic disease might be attributed to the physiological immunodeficiency of immaturity and factors regulating non-inflammatory Th1 profiles [54,55]. Paradoxically, a characteristic feature of neonatal T-cell responses is lower interferon γ (IFNγ) synthesis, a skewing toward a Th2 dominance, and generation of Th2-driven pro-inflammatory cytokine patterns (IL-4, -5, -9, -13) that propagate allergic inflammation [56]. This Th2 response leads to an influx of eosinophils in airways and immunoglobulin E (IgE) synthesis from B cells, and when combined with mucus hypersecretion and airway hyperresponsiveness, it leads to the onset of asthma symptoms [56]. This is important because both a Th1 phenotype and IFNγ—the main cytokine produced by Th1 cells—are implicated in the inhibition of the allergic march and, therefore, asthma [57]. The proposed mechanism by which a Th1 dominance confers a protective effect against allergy is by inhibiting Th2 cell-driven inflammation, the recruitment of eosinophils into airways, and mucus overproduction—key features of allergic inflammation [57]. With respect to T-cell proliferation, naïve CD4+ T cells and regulatory T cells (T reg) are key players in immune modulation and play a central role in the inflammatory process [42,58]. The fate of T cells depends on the direction of naïve CD4+ T-cell differentiation and maturation, favoring either a Th1 or Th2 profile and influencing either the propagation or the inhibition of the allergic march [42].

Alternatively, the lower capacity of neonatal T cells to express protein kinase C Ζeta (PKCζ) and a deficiency in PKC-dependent mitogen-activated protein kinases (MAPKs) cause dysregulation in T-cell division [59,60] and a greater tendency for the production of Th2 cytokines (IL-5, IL-13) [60], thus orchestrating a cascade of events favoring allergy development. D’Vaz et al., in an early study, demonstrated that diminished PKCζ expression in neonatal cord blood cells was associated with higher IL-13 responses at 6 months and lower IFNγ production by mature T cells and predicted the development and severity of allergic disorders in high-risk infants, including recurrent wheezing [61]. On the other side of the coin, fish oil supplementation in mothers during pregnancy was associated with increased levels of PKCζ via modification of histone acetylation at the PKCζ gene promoter region [61,62]. These data demonstrate that environmental exposures, including maternal diet, might regulate allergy susceptibility via the PKCζ gene. Potentially, fish oil supplementation or increased intake of fatty fish prenatally, could be a feasible allergy-preventive strategy in high-risk infants, having clinical effects in reducing allergic disease. Along the same line, the protective effects of vitamins D and A during the perinatal period in relevance to allergy have also been documented [63]. Strong evidence substantiates that vitamin D deficiency during pregnancy caused an imbalance in the Th1/Th2 ratio, decreased IFNγ production, but increased IL-4 concentration [63]. It is believed that maternal vitamin D deficiency influenced methylation status by regulating DNA methyltransferase (DNMT) activity—an enzyme participating in DNA methylation processes [63]. Interestingly, maternal vitamin D supplementation rectified these abnormalities [63]. In another study, vitamin D deficiency in the cord blood of infants contributed to an increased risk of allergic disease mediated by hypomethylation of microtubule-associated monooxygenase, calponin and LIM domain containing 3 (MICAL3), a gene responsible for the generation of reactive oxygen species [64]. Similarly, vitamin A, known for having immunomodulatory effects, was associated with lower Th17 and IgE responses and higher Foxp3+ T regulatory cells exerting chromatin structural modifications, epigenetic silencing of Th9 cells, and decreased histone acetylation B-cell promotor loci [43]. Experimental studies show that the imbalance between Th9 and Treg cells is closely related to asthma pathogenesis [65].

Given the above, one might speculate that dietary manipulation during this vulnerable period of fetal growth from conception until the first years of life—a critical window for epigenetic processes and programming—could lead to phenotypic alterations that are sustained throughout the lifespan, determine the fate of neonatal T-cell development and dominance, and ultimately affect allergic disease progression. More studies are urgently required to provide a proof of concept.

Another important finding in our study was that stratified analyses revealed stronger associations in specific subgroups. The association between first- and second-trimester folic acid supplementation and pre-adolescent asthma was stronger by approximately 50% in males, while the association with the third-trimester supplementation was stronger by 82% in children born prematurely (<37 weeks). Prematurity is a well-established risk factor for asthma development. For example, Zhang et al. conducted a cross-sectional analysis of over 90,000 children (<17 years) using data from the U.S. National Survey of Children’s Health (NSCH) (2011–2012), reporting that children born preterm had 64% higher odds of developing asthma compared with their full-term peers, even after adjusting for sociodemographic and environmental factors [66]. This further underscores the relevance of perinatal exposures in shaping long-term respiratory outcomes in children. In contrast to the present study, studies focused solely on full-term infants have also demonstrated that maternal folic acid supplementation during pregnancy is associated with increased asthma risk in offspring [24,25], further emphasizing the need to consider both the timing and the context of exposure.

In addition, AGA infants exhibited a significant association between first-trimester folic acid supplementation and asthma by 40% increased odds. Regarding the birth weight for gestational age on asthma risk in childhood, Wang et al. (2022) performed a meta-analysis exploring the impact of birth weight corrected for gestational age on asthma [67]. Data from twelve studies and over six million participants revealed that the SGAs and LGAs of infants were not associated with increased asthma risk as compared with the non-SGA or appropriate AGA group [67]. Paradoxically, our study found that AGA was associated with increased asthma odds. With respect to mode of delivery, a contemporary meta-analysis of thirty-five cohorts undertaken by Zhong et al., documented that offspring delivered by Cesarean section had 18% higher odds of asthma incidence as compared with delivery via the normal route [68].

With respect to environmental exposures, associations between folic acid supplementation during the third trimester and pre-adolescent asthma were stronger among non-smoking mothers by 52%, in those attending schools located in lower socioeconomic areas by 102%, and in neighborhoods having less traffic by 73% and more parks by 52%. The positive association between folic acid supplementation among pre-adolescents of non-smoking mothers may reflect epigenetically mediated transgenerational effects [19,45], whereas in smoking mothers, this association might be masked by the overriding effects of tobacco-related exposures [19]. A pooled analysis of eight birth cohort studies (*n* > 21,000) reported a 65% increased risk of asthma in preschoolers exposed to maternal smoking [69], while data from the ‘Japan Environment and Children’s Study (JECS)’ (*n* ≈ 75,000 mother–child dyads) found a 14% increased risk of asthma with passive smoking and a 34% increased risk with active smoking during pregnancy [39]. These effects may be mediated by epigenetic changes, such as DNA methylation and histone modifications [45,70]. Overall, the findings of this study support the original hypothesis that perinatal and environmental factors modify the association between maternal folic acid supplementation during pregnancy and pre-adolescent asthma and are substantiated by evidence from the literature [66]. These risk factors are representative of the complex interplay between genes and environmental exposures that influence fetal lung development and immunologic responses, thereby leading to asthma in later life [17,19].

More specifically, the positive association between maternal folic acid intake and asthma in pre-adolescents attending schools in low socioeconomic regions and residing in neighborhoods with less traffic is possible for a variety of reasons—namely, socioeconomic disparities [5,71]. Taken together, conditions of poverty [5], poor housing and living conditions [5], unhealthy diets [72], and limited access to medical and health care centers [16] are some parameters linking asthma in pre-adolescents belonging to vulnerable families. On the other hand, the finding of increased asthma odds in neighborhoods with parks might be explained by increased exposure to pollen, a common asthma trigger [73].

To sum up, these results are consistent with the theory of perinatal synergistic mechanisms, which suggests that perinatal factors alone have little or no effect on asthma development and confer a slight increase in risk. For this reason, the risk associated with folic acid supplementation during pregnancy may be amplified when it interacts with other perinatal or environmental exposures rather than acting as an isolated factor.

### Strengths and Limitations

To the best of our knowledge, this is the first study identifying/elucidating perinatal and environmental factors that strengthen the association between maternal folic acid supplementation and asthma in pre-adolescents and specifically in the Hellenic pediatric population. To date, there exists a multitude of studies focusing on the impact of maternal supplementation during pregnancy and neural tube defects in offspring [74]. Hence, this study is important in providing novel insights on respiratory outcomes, in extending the existing literature beyond neurological disorders, and in contributing valuable data to the field of pediatric respiratory health.

From a statistical point of view, prior studies have examined the impact of maternal folic acid supplementation in pediatric asthma, adjusting for a few covariates (such as maternal age at delivery, parity, child’s sex, birth weight, education, and maternal race) [75,76]. We also controlled for a range of potential perinatal and environmental risk factors for asthma collated from research studies [31,32,33], thus minimizing the likelihood of bias in the results and strengthening the validity and credibility of our findings. Another forte is that schools were selected from municipalities located in lower, medium, and higher socioeconomic areas; hence, our findings could be extrapolated to and representative of the national Hellenic population. However, it is important to acknowledge that women at risk of preterm birth may be more likely to receive supplements. Additionally, we lacked data on family history of asthma, which may have led to residual confounding.

Our study offers three key innovations. First, it tests a novel hypothesis grounded in the Barker theory of developmental origins of health and disease, specifically, ‘the impact of perinatal and environmental stimuli during intrauterine life on the association of maternal folic acid supplementation and asthma outcome,’ [45]. Second, unlike most previous studies targeting asthma during infancy and early childhood [77], our research addresses the understudies’ transitional period between childhood and adolescence. With the onset of puberty, pre-adolescence is a critical period of physical, psychological, and social development [78]. Biologically, the surge in female sex hormones during this age primes the background/setting for asthma development in adolescent females and women [37]. Third, rather than assessing supplementation across the entire pregnancy as a single exposure, the study examined its effects during distinct and critical windows of fetal development—namely the first, second, and third trimesters. This approach allows for a more nuanced understanding of the timing-specific influences of folic acid on asthma risk and helps identify potential sensitive periods of susceptibility. Given the well-established critical window for folic acid to prevent neural tube defects—specifically spina bifida—in early gestation (400 µg/day starting ≥1 month before conception through the first 2–3 months) [79], it becomes clinically relevant to evaluate trimester-specific supplementation. By continuing folic acid during early pregnancy and then discontinuing supplementation in later trimesters, one might still confer the protective benefit against neural tube defects, while potentially reducing the asthma risk linked to later-stage folic acid exposure. The analyses by trimester thus support exploring whether a targeted cessation of supplementation after the neurulation period could optimize both neural tube and respiratory outcomes.

On the other hand, this study has certain limitations. A weakness of cross-sectional studies is the inability to determine a causal inference of maternal folic acid supplementation and pre-adolescent asthma [80]. However, our study is useful in setting the foundations for new hypotheses to be addressed in well-designed longitudinal studies in a variety of settings. A downfall of this study was that folic acid supplementation and asthma were assessed qualitatively using questionnaires, which are prone to subjectivity, reporting or recall bias, and under- or over-reporting of the exposure [81]. Ideally, objective biomarkers on folic acid status or data on the exact quantity of folic acid supplemented to mothers provide a more accurate estimation. Spirometry is the gold standard of pulmonary function tests that is accurate in assessing pulmonary mechanics and in diagnosing asthma in children and adolescents [82]. Moreover, the associations reported are modest and often borderline significant and should be interpreted with caution. Alternatively, in parents, a lack of recognizing symptoms as characteristic features of asthma might result in information bias. Other possible sources of confounding not accounted for, such as the Tanner stages of sexual maturity [78], maternal intake of vitamin D [83], frequency of respiratory infections in children [84], BMI [85], family history of allergic diseases (rhinitis and eczema) [86], and a potential recall bias of folic acid supplementation intake considering the time elapsed between birth and pre-adolescence, are worth further exploration.

## 5. Conclusions

Maternal folic acid supplementation, particularly during the later trimesters, was modestly associated with increased odds of pre-adolescent asthma, with potential modification by perinatal and environmental factors. While these findings are exploratory, they underscore the need for future research to assess whether the timing of folic acid exposure during pregnancy may differentially influence asthma risk.

## Figures and Tables

**Table 1:** Characteristics of participants by pre-adolescent asthma status (N = 2332).

Characteristics		Pre-Adolescent Asthma		
		No *n* (%)	Yes *n* (%)	*p*-Value
**Demographic**				
**Age (years), *n* (Mean ± SD)**		1876 (11.2 ± 0.7)	450 (11.1 ± 0.6)	**0.040** ^b^
**Child’s Sex, *n* (%)**	Males	892 (47.4)	263 (58.3)	**<0.001** ^a^
	Females	989 (52.6)	188 (41.7)	
**Socioeconomic Level of School (SEL), *n* (%)**	Lower	498 (26.5)	106 (23.6)	**0.017** ^a^
	Medium	638 (34.0)	133 (29.6)	
	Higher	743 (39.5)	211 (46.8)	
**Mothers educational level, *n* (%)**	Primary	127 (7.4)	24 (5.9)	0.47 ^a^
	Secondary	845 (49.3)	211 (51.6)	
	Tertiary	742 (43.3)	174 (42.5)	
**Maternal age (years)** **[Median, (min, max) IQR]**		[40 (26, 58) IQR: 32]	[40 (28, 58) IQR: 30]	**0.002** ^c^
**Environmental**				
**Neighborhood has park areas for exercise, *n* (%)**	Agree	694 (46.6)	175 (48.1)	0.60 ^a^
	Disagree	797 (53.5)	189 (51.9)	
**Neighborhood has too much traffic, *n* (%)**	Agree	1062 (68.9)	236 (64.5)	**0.042** ^a^
	Disagree	456 (31.1)	130 (35.5)	
**Perinatal**				
Maternal pre-pregnancy weight, *n* (%)	UW/NW	1525 (81.1)	375 (83.1)	0.345 ^a^
	OW/OB	356 (18.9)	76 (16.8)	
**Gestational diabetes, *n* (%)**	Yes	41 (2.2)	17 (3.8)	**0.047** ^a^
	No/I don’t know	1836 (97.8)	428 (96.2)	
**Mode of delivery, *n* (%)**	Normal Birth	1366 (72.6)	299 (66.3)	**0.008** ^a^
	Cesarean	515 (27.4)	152 (33.7)	
**Gestational age, *n* (%)**	<37 weeks	339 (18.0)	99 (21.9)	0.05 ^a^
	≥37 weeks	1542 (82.0)	352 (78.1)	
**Weight categories for gestational age, *n* (%)**	AGA	1528 (81.2)	347 (76.9)	0.12 ^a^
	SGA	219 (11.6)	65 (14.4)	
	LGA	134 (7.1)	39 (6.7)	
**Exclusive breastfeeding, *n* (%)**	Not Exclusive	1724 (91.6)	418 (92.7)	0.47 ^a^
	Exclusive	157 (8.4)	33 (7.3)	
**Maternal folic acid intake during pregnancy**				
**Trimester 1, *n* (%)**	No	1594 (84.7)	365 (81.1)	0.059 ^a^
	Yes	287 (15.3)	85 (18.9)	
**Trimester 2, *n* (%)**	No	1487 (79.1)	337 (74.9)	0.054 ^a^
	Yes	394 (20.9)	113 (25.1)	
**Trimester 3, *n* (%)**	No	1513 (80.4)	344 (76.4)	0.059 ^a^
	Yes	368 (19.6)	106 (23.6)	
**Mother smoking during pregnancy, *n* (%)**	No	1578 (83.9)	381 (84.5)	0.76 ^a^
	Yes	303 (16.1)	70 (15.5)	
**Passive smoking during pregnancy, *n* (%)**	No	1408 (74.9)	317 (70.3)	**0.047** ^a^
	Yes	473 (25.1)	134 (29.7)	

Bold text: statistically significant at 0.05. The discrepancy in the number of participants per variable was due to missing data. The *p*-value was estimated using the ^a^ Chi-square test, ^b^ Independent Sample *t*-test, and ^c^ Mann–Whitney U test. Key: UW/NW = Underweight/Normal weight. OW/OB = Overweight/Obese. SGA = Small for gestational age. AGA = Appropriate for gestational age. LGA = Large for gestational age. Min = Minimum. Max = Maximum. IQR = Interquartile range.

**Table 2:** Association between maternal folic acid supplementation during pregnancy and pre-adolescent asthma overall and stratified by child’s sex.

Maternal Folic Acid Intake (Yes)		Pre-Adolescent Asthma				
	*n*	cOR (95% CI), *p*-Value		*n*	aOR (95% CI), P_adj_ *	
1st Trimester	372	1.29 (0.99, 1.69), 0.059		322	1.32 (0.99, 1.76), 0.063	
2nd Trimester	507	1.27 (0.99, 1.61), 0.055		445	1.30 (0.99, 1.68), 0.051	
3rd Trimester	474	1.27 (0.99, 1.62), 0.059		411	**1.34 (1.03, 1.75), 0.030**	
**Stratified analysis by child’s sex**						
		**cOR (95% CI), *p*-Value**			aOR (95% CI), P_adj_ *	
**Strata**	***n***	**Male**	**Female**		**Male**	**Female**
1st Trimester	2066	1.42 (1.00, 2.01), 0.050	1.11 (0.73, 1.70), 0.62		**1.57 (1.07, 2.29), 0.018**	1.00 (0.62, 1.61), 0.99
2nd Trimester	2066	1.36 (0.99, 1.86), 0.053	1.09 (0.74, 1.59), 0.67		**1.50 (1.07, 2.11), 0.018**	1.01 (0.66, 1.54), 0.96
3rd Trimester	2066	1.29 (0.93, 1.77), 0.12	1.18 (0.80, 1.73), 0.41		1.36 (0.96, 1.93) 0.09	1.27 (0.84, 1.93), 0.26

Bold text: *p*-values statistically significant at 0.05. Key: cOR = Crude odds ratio. CI = Confidence interval. aOR = Adjusted odds ratio. Reference group 0 = No asthma. * P_adj_ = *p*-value estimated from the regression model adjusted for child’s sex, maternal education level, breastfeeding exclusive, passive smoking during pregnancy, pre-pregnancy weight, maternal age, gestational diabetes, pregnancy weight gain, and mode of delivery.

**Table 3:** Association between maternal folic acid supplementation during pregnancy and pre-adolescent asthma stratified by perinatal characteristics.

		Pre-Adolescent Asthma					
		cOR (95% CI), *p*-Value			aOR (95% CI), P_adj_ *		
**By Gestational Age**							
**Maternal Folic Acid Intake (Yes)**	***n***	**Gestational Age** **< 37 Weeks**	**Gestational Age** **≥ 37 Weeks**		**Gestational Age** **< 37 Weeks**	**Gestational Age** **≥ 37 Weeks**	
1st Trimester	2066	1.25 (0.69, 2.27), 0.45	1.31 (0.97,1.76), 0.08		1.46 (0.77, 2.77), 0.25	1.29 (0.94, 1,80), 0.12	
2nd Trimester	2066	1.12 (0.65, 1.92), 0.69	**1.31 (1.00, 1.71), 0.048**		1.25 (0.69, 2.25), 0.45	1.33 (0.99, 1.77), 0.058	
3rd Trimester	2066	1.44 (0.83, 2.47), 0.19	1.24 (0.94,1.63), 0.13		**1.82 (1.01, 3.27), 0.046**	1.28 (0.95, 1.73), 0.11	
**By Weight for Age**							
		**cOR (95% CI), *p*-Value**			aOR (95% CI), P_adj_ *		
**Maternal Folic Acid Intake (Yes)**	***n***	**Weight for Age = AGA**	**Weight for Age = SGA**	**Weight for Age = LGA**	**Weight for Age = AGA**	**Weight for Age = SGA**	**Weight for Age = LGA**
1st Trimester	2066	**1.39 (1.03, 1.88),** **0.029**	0.87 (0.40, 185), 0.71	1.21 (0.44, 3.30), 0.71	**1.41 (1.02, 1.95),** **0.036**	1.02 (0.45, 2.34), 0.96	0.85 (0.24, 2.99), 0.80
2nd Trimester	2066	1.24 (0.94, 1.63), 0.12	1.14 (0.61, 2.13), 0.68	1.81 (0.77, 4.27), 0.17	1.24 (0.92, 1.68), 0.15	1.41 (0.72, 2.76), 0.32	1.84 (0.65, 5.20), 0.25
3rd Trimester	2066	1.20 (0.90, 1.59), 0.21	1.36 (0.73, 2.53), 0.33	1.72 (0.74, 4.03), 0.21	1.24 (0.92, 1.69), 0.16	1.76 (0.90, 3.42), 0.09	1.37 (0.47, 3.95), 0.56

Bold text: statistically significant at 0.05. Key: CI = Confidence interval. aOR = Adjusted odds ratio. SGA = Small for gestational age. AGA = Appropriate for gestational age. LGA = Large for gestational age. Reference group 0 = No asthma. * P_adj_ = *p*-value estimated from the regression model adjusted for maternal education level, breastfeeding exclusive, passive smoking during pregnancy, pre-pregnancy weight, maternal age, gestational diabetes, pregnancy weight gain, and mode of delivery.

**Table 4:** Association between maternal folic acid supplementation during pregnancy and pre-adolescent asthma stratified by environmental and external factors.

		Pre-Adolescent Asthma								
		cOR (95% CI), *p*-Value					aOR (95% CI), P_adj_ *			
**Maternal Smoking**										
**Maternal Folic Acid Intake (Yes)**	***n***	**Maternal Smoking = No**		**Maternal Smoking = Yes**			**Maternal Smoking = No**		**Maternal Smoking = Yes**	
1st Trimester	2066	1.31 (0.97, 1.75), 0.073		1.23 (0.64, 2.39), 0.53			1.37 (1.00, 1.88), 0.050		1.12 (0.52, 2.40), 0.77	
2nd Trimester	2066	**1.35 (1.04, 1.75), 0.025**		0.92 (0.51, 1.69), 0.80			**1.44 (1.08, 1.91), 0.012**		0.80 (0.40, 1.57), 0.51	
3rd Trimester	2066	**1.36 (1.03, 1.77), 0.027**		0.91 (0.48, 1.70), 0.76			**1.52 (1.14, 2.02), 0.005**		0.80 (0.40, 1.61), 0.53	
**School Economic Level**										
		**cOR (95% CI), *p*-Value**					aOR (95% CI), P_adj_ *			
**Maternal Folic Acid Intake (Yes)**	***n***	**SEL = Low**	**SEL = Medium**			**SEL = High**	**SEL = Low**	**SEL = Medium**		**SEL = High**
1st Trimester	2066	**1.84 (1.05, 3.21), 0.034**	1.02 (0.61, 1.70), 0.95			1.23 (0.84, 1.80), 0.29	**1.94 (1.07, 3.55), 0.030**	1.03 (0.59, 1.81), 0.92		1.26 (0.83, 1.93), 0.27
2nd Trimester	2066	**1.68 (1.03, 2.77), 0.039**	1.10 (0.70, 1.72), 0.69			1.17 (0.83, 1.66), 0.37	**1.87 (1.10, 3.19), 0.021**	1.11 (0.68, 1.83),0.67		1.17 (0.80, 1.71), 0.42
3rd Trimester	2066	**1.73 (1.04, 2.85), 0.034**	1.01 (0.64, 1.61), 0.95			1.22 (0.86, 1.75), 0.27	**2.02 (1.17, 3.49), 0.011**	1.10 (0.66, 1.82), 0.72		1.29 (0.88, 1.91), 0.19
**Neighborhood Parks**										
**Maternal Folic Acid Intake (Yes)**	***n***	**Neighborhood Parks = Agree**			**Neighborhood Parks = Disagree**		**Neighborhood Parks = Agree**		**Neighborhood Parks = Disagree**	
		**cOR (95% CI), *p*-Value**					aOR (95% CI), P_adj_ *			
1st Trimester	1664	1.15 (0.75, 1.77), 0.511			**1.61 (1.08, 2.40), 0.020**		1.21 (0.76, 1.93), 0.413		**1.60 (1.04, 2.49), 0.034**	
2nd Trimester	1664	1.41 (0.97, 2.05), 0.069			1.18 (0.82, 1.72), 0.369		1.41 (0.94, 2.12), 0.098		1.24 (0.84, 1.85), 0.282	
3rd Trimester	1664	**1.52 (1.04, 2.23), 0.031**			1.09 (0.74, 1.59), 0.673		**1.52 (1.00, 2.30), 0.047**		1.18 (0.78, 1.79), 0.422	
**Neighborhood Traffic**										
**Maternal Folic Acid Intake (Yes)**	***n***	**Neighborhood Traffic = Agree**			**Neighborhood Traffic = Disagree**		**Neighborhood Traffic = Agree**		**Neighborhood Traffic = Disagree**	
		**cOR (95% CI), *p*-Value**					aOR (95% CI), P_adj_ *			
1st Trimester	1694	1.28 (0.89, 1.84), 0.187			1.57 (0.96, 2.56), 0.073		1.32 (0.89, 1.98), 0.172		1.55 (0.92, 2.63), 0.103	
2nd Trimester	1694	1.31 (0.95, 1.81), 0.103			**1.63 (1.05, 2.52), 0.029**		1.39 (0.97, 1.98), 0.069		1.52 (0.95, 2.45), 0.081	
3rd Trimester	1694	1.28 (0.92, 1.79), 0.143			**1.74 (1.11, 2.72), 0.016**		1.37 (0.96, 1.97), 0.086		**1.73 (1.07, 2.82), 0.026**	

Bold text: statistically significant at 0.05. Key: CI = Confidence interval. aOR = Adjusted odds ratio. SEL = School economic level. cOR = Crude odds ratio. Reference group 0 = No asthma. * P_adj_ = *p*-value estimated from the regression model adjusted for maternal education level, breastfeeding exclusive, passive smoking during pregnancy, pre-pregnancy weight, maternal age, gestational diabetes, pregnancy weight gain, and mode of delivery.

## Data Availability

The original contributions presented in this study are included in the article/Supplementary Materials. Further inquiries can be directed to the corresponding author.

## Supplementary Materials

The following supporting information can be downloaded at: https://www.mdpi.com/article/10.3390/nu17182989/s1, S1 HGS Parental Questionnaire; Figure S1: Directed Acyclic Diagram describing the causal-effect of the association between maternal folate intake during the 3rd trimester and pre-adolescent asthma.

## Abbreviations

The following abbreviations are used in this manuscript:

AGA	Appropriate for gestational age
95% CI	95% Confidence interval
HGS	Healthy Growth Study
ISAAC	International Study on Asthma and Allergy in Childhood
IQR	Interquartile range
LGA	Large for gestational age
OR	Odds ratio
SGA	Small for gestational age
WHO	World Health Organization
