# Supplementary material for: Maternal Folic Acid Supplementation, Perinatal Factors, and Pre-Adolescent Asthma: Findings from the Healthy Growth Study
Source: Nutrients. 2025 Sep 18;17(18):2989. doi: 10.3390/nu17182989 (PMC12472973; doi:10.3390/nu17182989)
Supplement: Supplementary file 1 [file nutrients-17-02989-s001.zip › 1. Fig S1 DAG 2.9.25MP use.pdf]

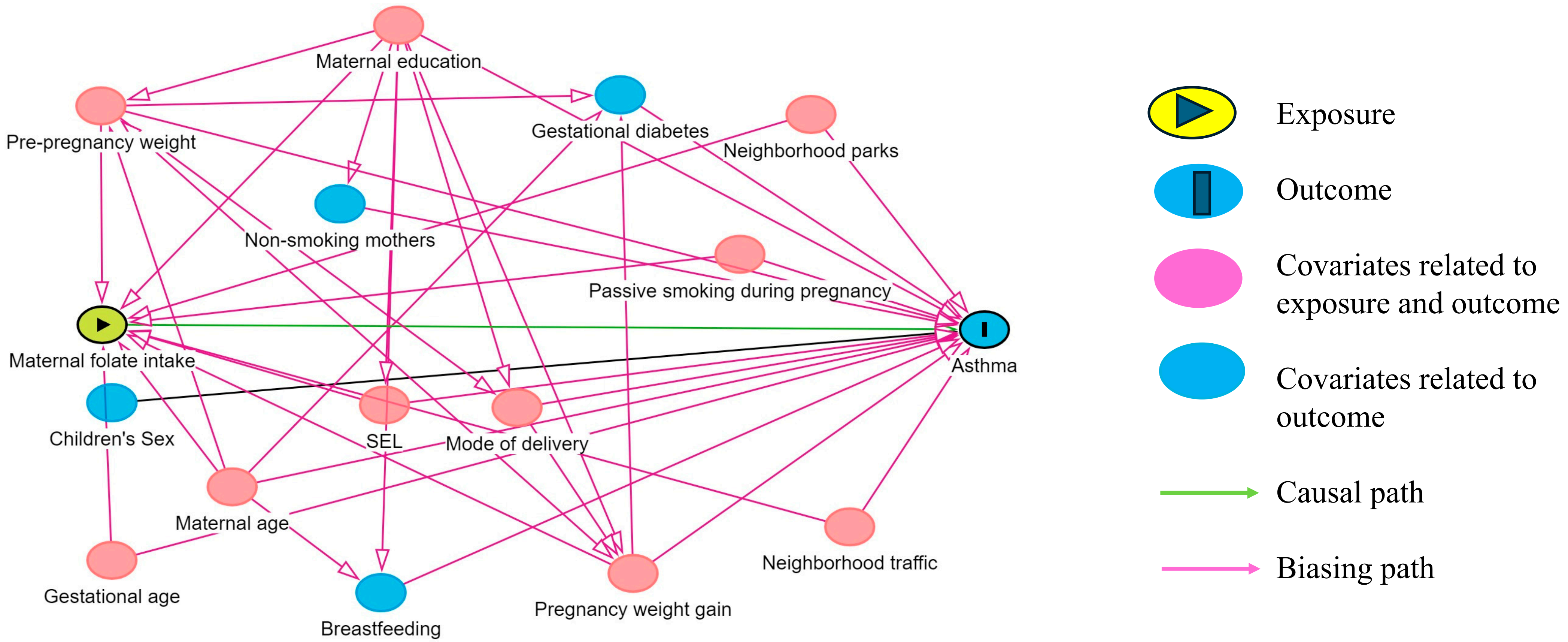

Figure S1. Directed Acyclic Diagram describing the causal-effect of the association between maternal folate intake during the 3rd trimester and pre-adolescent asthma

**This diagram was constructed using DAGitty v3.1 available from https://www.dagitty.net/dags.html

Ref: Textor J, van der Zander B, Gilthorpe MK, Liskiewicz M, Ellison G. Robust causal inference using directed acyclic graphs: the R package 'dagitty'. Internat J Epidemiol. 45(6):1887-1894, 2016.
