# Supplementary material for: Maternal Folic Acid Supplementation, Perinatal Factors, and Pre-Adolescent Asthma: Findings from the Healthy Growth Study
Source: Nutrients. 2025 Sep 18;17(18):2989. doi: 10.3390/nu17182989 (PMC12472973; doi:10.3390/nu17182989)
Supplement: Supplementary file 1 [file nutrients-17-02989-s001.zip › 1. Supplemental S1 HGS Parental questionnaire ENG 3.9.25MP.pdf]

HGS Parental Questionnaire

CHILD'S DATE OF BIRTH: ____/_____/___

CHILD ID.:

RELATIONSHIP OF PERSON COMPLETING THE QUESTIONNAIRE (e.g. mother, father, grandmother etc.):

____

DATE THAT THE QUESTIONNAIRE WAS COMPLETED: _____ / _____ / _____

Please provide us with as much of the following information as you know. We ensure that all the information you provide below is strictly confidential

A. CHILD'S MEDICAL HISTORY

**PREGNANCY**			
Mother's pre-pregnancy weight (2-3 months before conception)			
How much weight (kg) did the mother put on during pregnancy?			
Duration of pregnancy (in weeks)			
Type of pregnancy (Please circle)	Normal	Caesarean	
**Indicate with a tick √ at which trimester of pregnancy the mother practiced any of the following.**			
	1st trimester	2nd trimester	3rd trimester
Use of folic acid supplements (mothers before pregnancy……..)			
Use of iron supplements			
Smoked during pregnancy (No. cigarettes/day)			
Passive smoking at work or home (How many cigarettes were smoked in that area)			
Alcohol consumption during pregnancy (servings/ day)			
Smoking during breastfeeding (No. Cigarettes/day)			
Alcohol consumption during breastfeeding (servings /day)			

To be completed from the child's health booklet:

**INFANT**	**Weight (kg)**	**Height (m)**	**Head Circumference (cm)**
At birth			
1st month			
2nd month			
3rd month			
4th month			
5th month			
6th month			
7th month			
8th month			
9th month			
10th month			
11th month			
12th month			

Did the mother have high blood pressure or gestational diabetes during pregnancy?

1. High blood pressure? Yes / No/ I don't know
2. Gestational diabetes? Yes /No/I don't know

B. BREASTFEEDING AND FEEDING DURING THE FIRST 12 MONTHS OF THE CHILD'S LIFE

Indicate with a tick √ the months during which your child was breastfed…

	Birth	1st	2nd	3rd	4th	5th	6th	7th	8th	9th	10th	11th	12th
Exclusive breastfeeding													
Mixed feeding (Infant formula and breastmilk)													

Indicate with a tick √ the months during which your child received…….

	Birth	1st	2nd	3rd	4th	5th	6th	7th	8th	9th	10th	11th	12th
Milk substitute (i.e. Commercial infant milk powder)													
Tea, Chamomile (e.g for stomach pain)													
Liquid foods (e.g diluted fruit juice)													
Solid and semi-solid foods (π.χ. Farina Lacte, vanilla custard, custard with fruits)													

C. PARENTAL DEMOGRAPHIC AND SOCIO-ECONOMIC DETAILS

		Age	***Weight*** (κιλά)	***Height*** *(m)*	*Origin*	*Education (years)*	*Occupation*	*Employed*	*Retired*
Child's Father								**Yes No**	**Yes No**
Child's Mother								**Yes No**	**Yes No**
Siblings	1								
	2								
	3								

2. Please answer these questions if you smoke:

	Total number of cigarettes per day	*Number of cigarettes smoked in the home per day*	*Number of years smoking*
Child's Father			
Child's Mother			

3. What is your average annual family income over the last 3 years?

☐ <12.000 € per year ☐ 12. 000-20.000 € per year ☐ 20.000-30.000 € per year ☐ 30.000-40.000 € per year

☐ 40.000-50.000 € per year ☐ 50.000-60.000 € per year ☐ 60.000-70.000 € per year ☐ 70.000-80.000 € per year

☐ 80.000-90.000 € per year ☐ >90.000 € per year

4. Please indicate the number of cars in your family: ………………………………………

5. Do you live in your own home? ☐ Yes ☐ No

6. How many square meters is the house or apartment you live in? ………………….m^2

7. What is your marital status?

☐ Unmarried ☐ Married ☐ Divorced ☐ Widow

8. Who else regularly takes care of your children besides you?

☐ Grandfather ☐ Grandmother ☐ Babysitter ☐ Other……………………………….

9. In total, how may persons live in your house? ………

Who? ………………………………….. …………………………………..

…………………………………... …………………………………...

……………………………………. …………………………………...
